# Metagenomic analysis of the camel rumen archaeome and its functional potential

**DOI:** 10.3389/fvets.2025.1738018

**Published:** 2026-01-23

**Authors:** Mohamed Abdelmegeid, Mohamed Zeineldin, Rabiha Seboussi, Mahmoud Mohamadin, Abdulrahman S. Alharthi, Nabil Mansour, Lamiaa A. Okasha, Ahmed A. Elolimy, Eva-Maria Saliu

**Affiliations:** 1 College of Veterinary Medicine, University of Al Dhaid, Sharjah, United Arab Emirates; 2 Department of Animal Medicine, Faculty of Veterinary Medicine, Kafrelsheikh University, Kafrelsheikh, Egypt; 3 Department of Animal Medicine, Faculty of Veterinary Medicine, Benha University, Benha, Egypt; 4 Department of Animal Production, College of Food and Agriculture Sciences, King Saud University, Riyadh, Saudi Arabia; 5 Center of Excellence in Biotechnology Research, King Saud University, Riyadh, Saudi Arabia; 6 Department of Camel Research, Fujairah Research Centre (FRC), Fujairah, United Arab Emirates; 7 Department of Theriogenology, Faculty of Veterinary Medicine, Kafrelsheikh University, Kafr El-Sheikh, Egypt; 8 Bacteriology Unit, Animal Health Research Institute AHRI, Agriculture Research Center ARC, Kafrelsheikh, Egypt; 9 Department of Integrative Agriculture, College of Agriculture and Veterinary Medicine, United Arab Emirates University, Al Ain, United Arab Emirates; 10 Department of Ecological Animal Husbandry, Faculty for Landscape Management and Nature Conservation, Eberswalde University for Sustainable Development, Eberswalde, Germany

**Keywords:** archaeome, camel, functional profiling, metagenomic, methanogens, microbial diversity

## Abstract

The camel rumen harbors a unique and underexplored archaeal community that plays a critical role in methanogenesis and ruminal fermentation. This study aimed to characterize the taxonomic composition and functional potential of the camel rumen archaeome using whole-genome shotgun metagenomic sequencing. Across the seven healthy racing camel rumen samples, the archaeal community was dominated by Euryarchaeota (50.1 ± 0.02%) and the Methanomada group (49.7 ± 0.03%), with *Methanobacteriaceae* and *Methanobrevibacter* representing the predominant family and genus, respectively. Species-level analysis revealed *Methanobrevibacter* sp. *YE315* and *Methanobrevibacter millerae* as the most abundant archaeal species across all samples. Alpha-diversity analyses indicated a diverse and evenly distributed archaeal population in the camel rumen. Beta-diversity based on Bray–Curtis and Jaccard dissimilarities demonstrated strong similarity among samples, highlighting a conserved archaeal community structure across individuals. Core microbiome assessment (≥ 80% occurrence) identified seven dominant *Methanobrevibacter* species as the stable core archaeome. Functional profiling revealed a consistent metabolic repertoire dominated by methanogenesis (PWY-5209), amino acid biosynthesis, and nucleotide metabolism pathways. Functional alpha-diversity metrics and beta-diversity clustering highlighted low inter-sample variability and a stable functional architecture. Overall, the camel rumen archaeome exhibited a stable and conserved community composition and functional architecture, underscoring its central role in hydrogen utilization and methane production within the rumen ecosystem. Although based on a small number of animals from a single location and therefore descriptive in nature, this study provides a comprehensive metagenomic overview of the taxonomic and functional profiles of the camel rumen archaeal community.

## Introduction

Methane (CH4) is a potent greenhouse gas, with a global warming potential over 25 times greater than that of carbon dioxide when evaluated over a 100-year period (1). A significant portion of anthropogenic methane emissions originates from enteric fermentation in ruminant livestock (2). While extensive research has been conducted on methane production in cattle and sheep, relatively little is known about methane emissions from dromedary camels (*Camelus dromedarius*), particularly those reared in arid and semi-arid regions such as the United Arab Emirates (UAE) (3). Despite their unique adaptation to harsh climates and their increasing importance in food security, the microbial drivers of methane production in camels remain largely unexplored compared to that of cattle and small ruminants (4). Therefore, understanding the diversity, structure, and functional potential of the camel archaeome is crucial for elucidating its role in rumen metabolism and its contribution to methane emissions.

Methanogens, a group of archaea responsible for methane production through anaerobic fermentation, are integral members of the herbivore gut microbiome (5). These microorganisms play a critical ecological role by converting carbon dioxide, hydrogen, and methyl compounds into methane during the final step of anaerobic digestion (6). In ruminants, the majority of methane is produced in the foregut (rumen), with additional contributions from the hindgut (5, 6). However, camels possess a distinct digestive physiology that differs significantly from that of true ruminants, including differences in foregut anatomy, fermentation dynamics, and feed conversion efficiency (7). These differences warrant an in-depth investigation of their gut methanogen communities to better understand their contribution to enteric methane emissions (8). Recent studies using real-time PCR (qPCR) have provided initial insights into the archaeal populations inhabiting the camel gastrointestinal tract (9). Despite their utility in quantifying specific microbial taxa, qPCR-based approaches are limited by their reliance on known gene targets, primer specificity, and an inability to provide detailed community structure or functional insights (10). To overcome these limitations, high-throughput sequencing, shotgun metagenomics, and bioinformatics can be integrated to enable a deeper exploration of archaeal diversity. This approach provides an unbiased view of microbial communities and their functional potential (8, 11). They allow for the identification of both known and previously unclassified methanogens, as well as the reconstruction of their metabolic potential and ecological interactions within the gut ecosystem (12).

This study aims to use high throughput metagenomic sequencing to analyze the taxonomic composition, diversity and functional potential of methanogens in camel rumen samples.

## Materials and methods

### Animals and diet

Seven clinically healthy racing dromedary camels (*Camelus dromedarius*), aged 4–6 years, were selected from a private racing camel facility in Dubai, UAE. The animals were maintained under uniform management and feeding conditions. The diet consisted of a balanced concentrate mix and 6 mm pelleted feed (Pellet I) formulated to meet the nutritional requirements of athletic camels (Table 1). Both feed types supplied sufficient energy and fiber for optimal rumen fermentation and performance.

**Table 1:** Nutritional composition of the feed offered to racing dromedary camels included in this study.

**Feed type**	**Moisture**	**Crude ash**	**Crude protein**	**Ether extracts**	**Crude fiber**	**ADF**	**NDF**
6 mm Pellet I	9.43	9.81	16.77	3.17	NA	18.26	31.56
Concentrate mix	10.51	5.48	10.98	2.07	11	12.5	25.52

NA: Crude fiber value was not provided in the manufacturer's feed analysis, and the original feed batch was unavailable for retrospective measurement.

### Ruminal sample collection

Rumen contents were collected from each camel before morning feeding using a sterile flexible stomach tube (10). Approximately 500 mL of rumen fluid was obtained and filtered through two layers of sterile cheesecloth to remove large particles. The pH was measured immediately using pH indicator strips (range 4.0–7.0) to confirm rumen health status. Aliquots of the clarified rumen liquor were snap-frozen in liquid nitrogen on-site, transported to the laboratory on dry ice, and stored at −80 °C until DNA extraction.

### DNA extraction and quality assessment

Total microbial genomic DNA was extracted from 200 mg of rumen content using the QIAamp^®^ PowerFecal^®^ DNA Kit (Qiagen, USA), following the manufacturer's instructions (13). The quality and integrity of extracted DNA were assessed using a NanoDrop ND-1000 spectrophotometer (Thermo Fisher Scientific) and Agilent Fragment Analyzer 5400 with the Genomic DNA Analysis Kit (DNF-488). DNA concentration was then quantified using a Qubit Fluorometer (Thermo Fisher Scientific) and the Qubit dsDNA HS Assay Kit.

### Shotgun metagenomic sequencing

High-quality DNA was used for library preparation with the Novogene NGS DNA Library Prep Set (catalog no.PT004) according to the manufacturer's protocol. Libraries were assessed for quality and quantity using the KAPA Library Quantification Kit (Roche) and Qubit dsDNA HS Assay Kit. Library size distribution was evaluated using the Agilent 2100 Bioanalyzer with the Agilent High Sensitivity DNA Kit, and qualitatively with the Agilent Fragment Analyzer system using the Qualitative DNA Kit (DNF-915-K1000). Sequencing was performed on the Illumina NovaSeq X Plus platform, generating 150 bp paired-end reads (PE150) using a 300-cycle reagent kit.

### Bioinformatics and statistical analyses

#### Quality control and host read removal

Raw shotgun metagenomic reads obtained from camel rumen samples were first subjected to quality control and host read filtering prior to downstream analysis. Adapter sequences and low-quality bases were trimmed using Trimmomatic (14). Host-derived sequences were removed by aligning reads to the *Camelus dromedarius* reference genome (NCBI Assembly: GCF_036321535.1) using Bowtie2 with default parameters (15). Only unmapped reads were retained for downstream taxonomic and functional analyses. The proportion of reads aligning to the host genome was very low across all samples, ranging from 0.07% to 0.11%, indicating minimal host DNA carryover from rumen fluid collection. Following host read removal, an additional quality check was performed using FastQC (16) to verify read integrity and ensure that trimming and filtering procedures did not introduce biases.

#### Taxonomic and functional profiling

The filtered reads were analyzed using the bioBakery Whole-Genome Shotgun (WGS) pipeline implemented on the NIH Nephele platform (17–19). Taxonomic classification was performed with MetaPhlAn 4.0, which utilizes clade-specific marker genes to estimate relative abundances of archaeal taxa (20). Functional potential was assessed using HUMAnN 3.0, which maps quality-filtered reads to the UniRef90 and KEGG Orthology (KO) databases for pathway and gene family quantification (21). Relative abundance tables were generated for both taxonomic levels (phylum, family, genus and species) and functional profiles (KO identifiers and MetaCyc pathways).

#### Data filtering and archaeal-specific subsetting

To focus on the archaeal community, only taxa annotated within the domain *Archaea* were retained. HUMAnN functional tables were cross-referenced with the archaeal taxonomic profiles, and gene families and pathways linked to archaeal species were extracted using Python scripts. Relative abundance values were normalized to percentage values within each sample prior to downstream analyses.

### Alpha and beta diversity analyses

Alpha diversity metrics including Species richness, Shannon, Simpson, Evenness and Chao1 indices were computed using the scikit-bio package in Python to evaluate within-sample archaeal diversity (22). Beta diversity was calculated using both Bray–Curtis dissimilarity and Jaccard distance to assess between-sample community variation (23). Principal Coordinate Analysis (PCoA) was applied to visualize compositional dissimilarities, and hierarchical clustering heatmaps were generated to depict archaeal distribution patterns across samples (24).

### Functional diversity and core archaeal analyses

Functional diversity metrics (functional richness, Shannon and Simpson) were computed based on KO abundance profiles. The archaeal core species and core functional features were defined as those present in 80 % and 100 % of samples with a relative abundance threshold ≥0.01%, respectively. The prevalence and abundance of core functions were visualized using heatmaps generated in seaborn (25).

### Differential abundance and stability analyses

To assess archaeal stability across animals, mean relative abundance and coefficient of variation (CV) were calculated for each archaeal species (26). Taxa with high abundance and low CV were identified as stable core members. For exploratory differential abundance, taxa and gene families with high variation were highlighted for potential functional specialization among individuals.

### Visualization and statistical analysis

Descriptive statistical analyses were performed to summarize the archaeal taxonomic and functional metrics across all samples, including relative abundances, alpha diversity indices, beta diversity distances, and functional diversity indices expressed as mean ± SD. All computational analyses and visualizations were conducted in Python using the libraries pandas, numpy, matplotlib, seaborn, and scikit-bio. Heatmaps, prevalence curves, UpSet plots, and stability plots to depict abundance distributions, core community composition, taxa overlap, and inter-sample variability (27). Since all samples represented healthy camels without treatment grouping, no inferential statistical tests were applied; instead, results were summarized descriptively to reflect conserved features in the camel archaeal community.

## Results

### Overview of sequencing and data processing

A total of 14 paired-end shotgun metagenomic libraries were generated from seven camel rumen samples. Sequencing quality metrics from the Nephele platform indicated high-quality datasets with low duplication rates and consistent GC content across samples. The number of raw reads per replicate ranged from 46.9 million to 75.2 million (mean = 64.5 million reads per sample). The average GC content was approximately 48–50%, consistent with rumen-associated archaeal and bacterial genomes. Duplication rates were low, ranging between 16.4% and 20.4%, suggesting minimal amplification bias. Following read preprocessing, adapter trimming, and quality filtering, host-derived reads were removed by aligning sequences to the *Camelus dromedarius* reference genome. After host read removal and processing through the Nephele bioBakery WGS pipeline, archaeal reads were successfully identified in all samples. Across the seven camel rumen samples, the total number of archaeal reads ranged from 0.78 to 2.47 million per sample, with an average of approximately 1.75 ± 0.55 million reads. Despite differences in total sequencing depth, archaeal populations were consistently detected in all samples, reflecting the stable presence of methanogenic lineages within the rumen ecosystem.

### Taxonomic composition of the archaeal community

**Figure 1:**
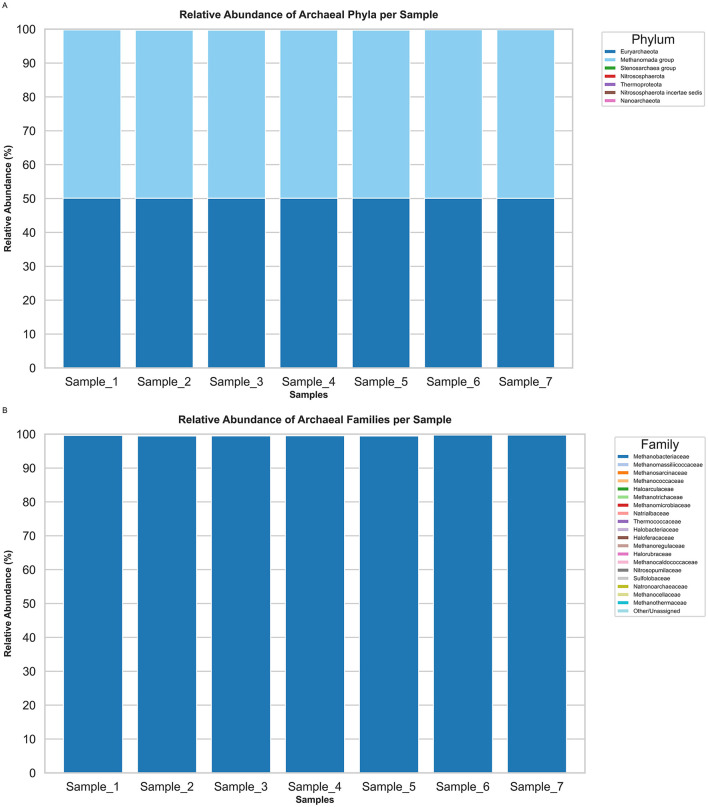
Phylum- and family-level composition of the archaeal community in camel rumen samples. **(A)** Relative abundance (%) of archaeal phyla across seven samples. **(B)** Relative abundance of archaeal families. Minor families are grouped as “Others/unassigned.”

**Figure 2:**
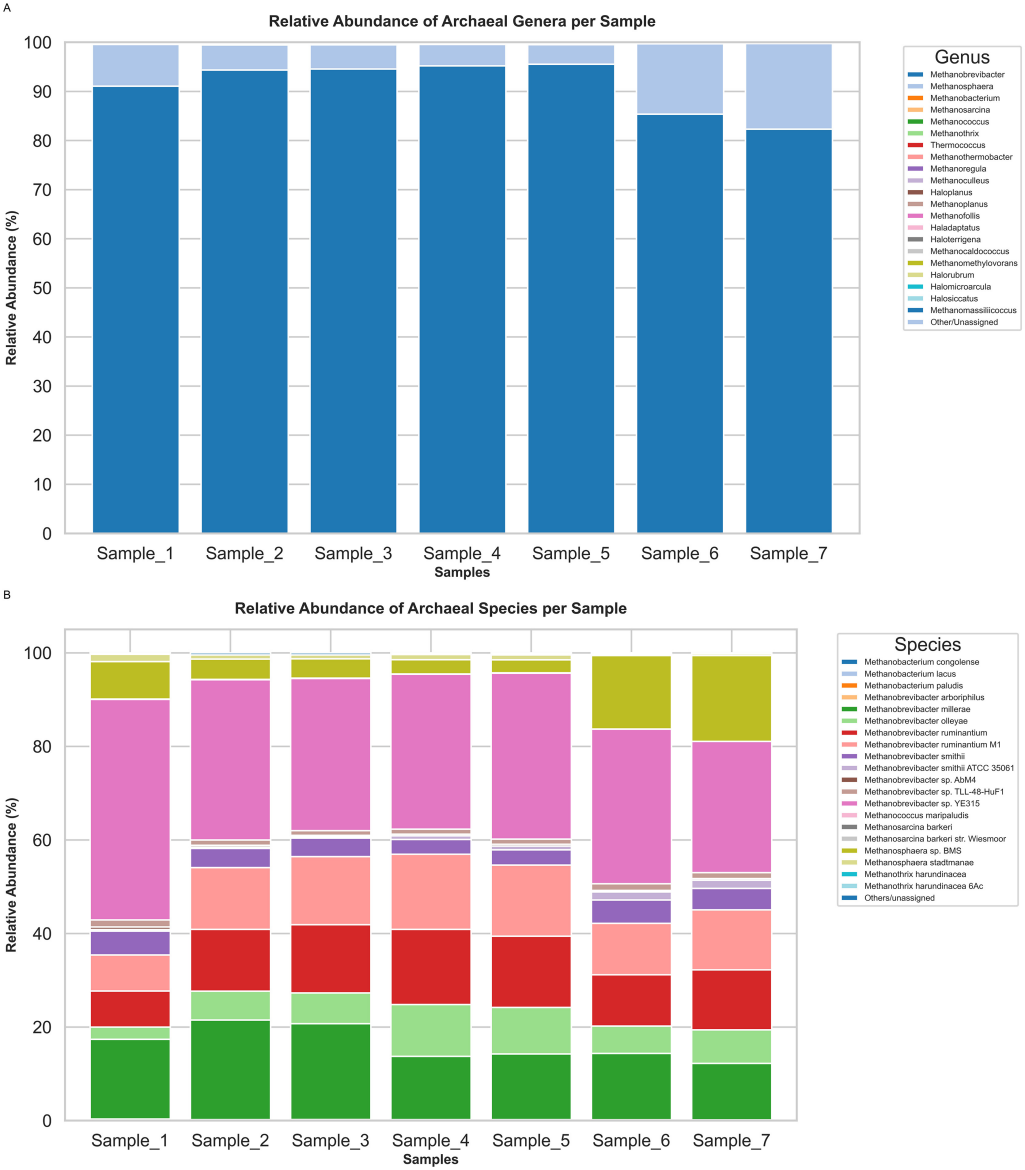
Genus- and species-level composition of the archaeal community in camel rumen samples. **(A)** Relative abundance (%) of archaeal genera. **(B)** Relative abundance of top species-level taxa. Minor genera and species are grouped as “Others/unassigned.”

At the phylum level, the archaeal community was remarkably consistent across all seven camel rumen samples (Figure 1A, Supplementary Table S1). The phylum Euryarchaeota dominated with a mean relative abundance of 50.10 ± 0.02 %, while the Methanomada group closely followed at 49.67 ± 0.08 **%**. All other archaeal phyla Stenosarchaea group (0.22 ± 0.06 %), Nitrososphaerota (0.005 ± 0.003 %), Thermoproteota (0.004 ± 0.002 %), Nitrososphaerota incertae sedis (0.001 ± 0.002 %), and Nanoarchaeota (undetectable or ≈ 0.000 %) collectively accounted for less than 1 % of archaeal reads in any sample. At the family level, the archaeal community in the camel rumen was dominated by the *Methanobacteriaceae*, which accounted for a mean relative abundance of 99.58 ± 0.09 % across the seven samples. The next most abundant families were *Methanomassiliicoccaceae* (mean = 0.062 ± 0.04 %) and *Methanosarcinaceae* (mean = 0.061 ± 0.03 %). All other archaeal families detected had individual mean abundances below ~0.03 % and collectively contributed less than 0.3 % of the total archaeal reads per sample (Figure 1B, Supplementary Table S1). At the genus level, archaeal community composition was strongly dominated by *Methanobrevibacter*, accounting for an average of 91.19 ± 3.13 % of the archaeal reads across all seven camel rumen samples. The second most abundant genus, *Methanosphaera*, comprised 8.38 ± 4.30 % of reads. All other archaeal genera individually contributed ≤ 0.12 % of reads and collectively represented less than 2 % of the archaeal community in any sample (Figure 2A, Supplementary Table S1). At the species level, archaeal community in the camel rumen was dominated by *Methanobrevibacter* sp. *YE315* and *Methanobrevibacter millerae*, which together accounted for approximately 50.64% ± 4.23% of total archaeal reads across samples. *Methanobrevibacter* sp. *YE315* averaged 34.81 ± 4.68% while *Methanobrevibacter millerae* averaged 16.1 ± 3.13% Secondary species such as *Methanobrevibacter ruminantium* (12.93 ± 2.09%) and *Methanosphaera* sp. *BMS* (8.06 ± 1.11%) were present at lower but consistent levels, while all remaining detected species together comprised less than 6% of the archaeal population in any individual sample (Figure 2B, Supplementary Table S1).

### Archaeal diversity and community structure

**Figure 3:**
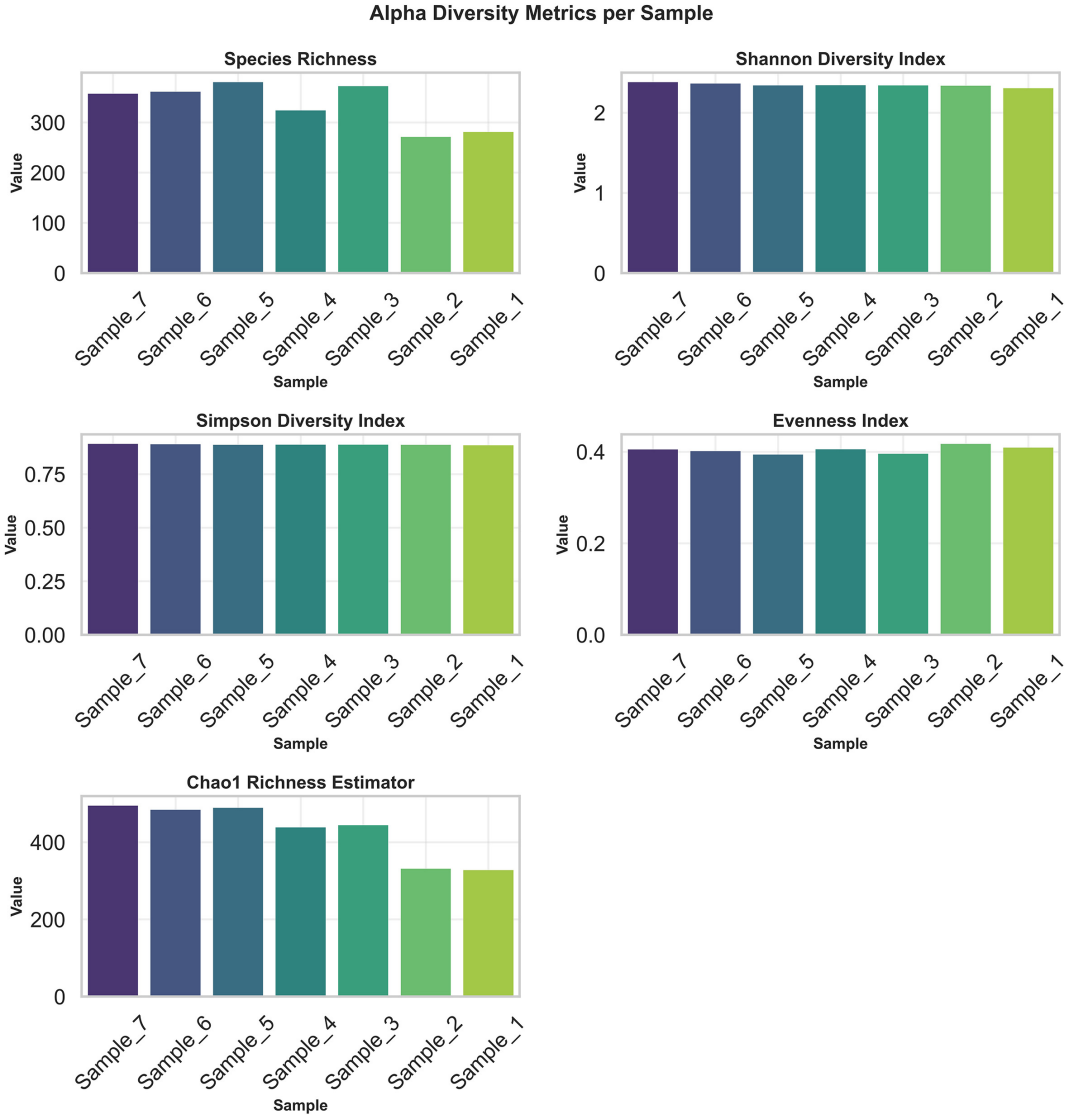
Within-sample archaeal diversity metrics (species richness, Shannon index, Simpson index, evenness and Chao1 richness) in camel rumen samples. Values represent mean ± SD.

Alpha-diversity analyses of the archaeal communities revealed consistent within-sample diversity across the seven camel rumen samples (Figure 3). Observed species richness ranged from 271 to 380 taxa, with a mean of 335.14 ± 39.1 across the seven camel rumen samples. The Shannon index averaged 2.346 ± 0.024, and the Simpson index was 0.887 ± 0.002 across the seven camel rumen samples. The evenness index averaged 0.404 ± 0.006, indicating a relatively uniform distribution of archaeal taxa, while the Chao1 richness estimator averaged 429.1 ± 68.5, reflecting high species richness across the seven camel rumen samples.

**Figure 4:**
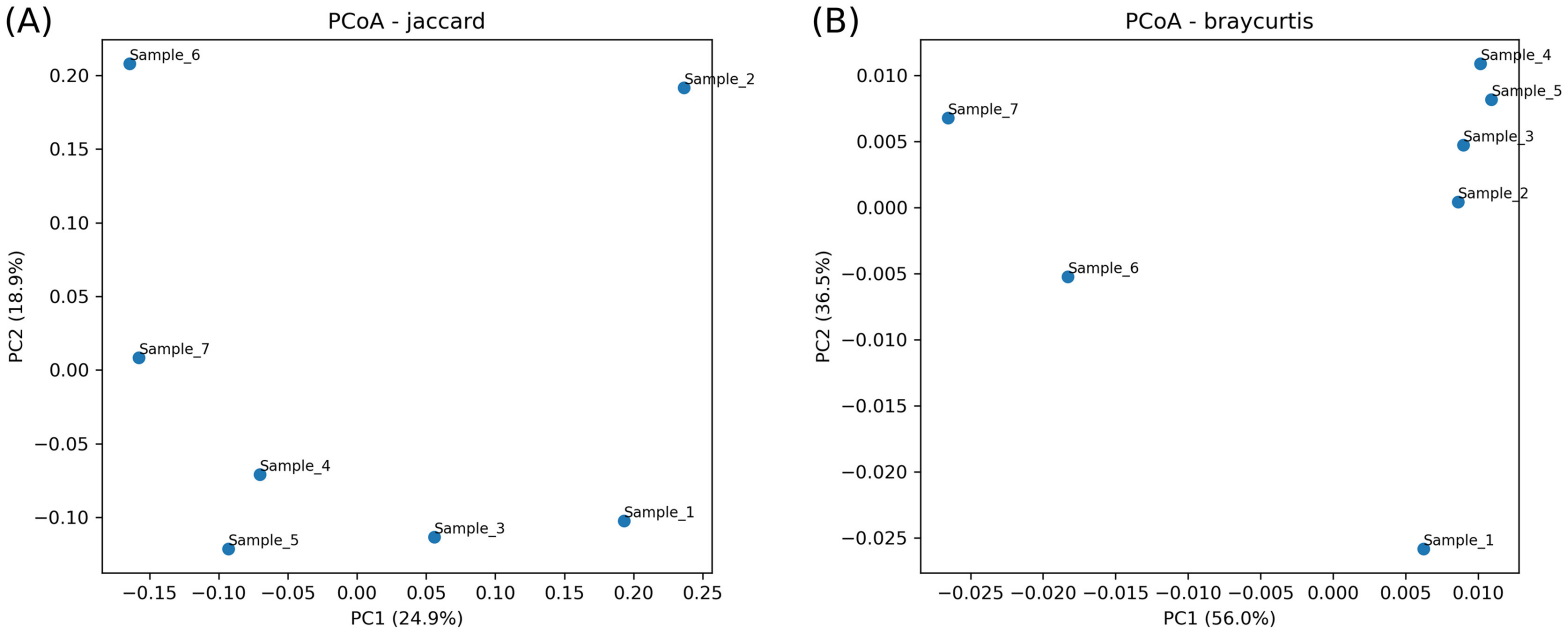
Principal Coordinates Analysis (PCoA) of archaeal community structure across camel rumen samples. **(A)** (Jaccard distance) illustrates presence/absence-based ordination. **(B)** (Bray–Curtis distance) depicts abundance-based ordination.

Between-sample community dissimilarities were assessed using PCoA based on Jaccard (presence/absence) and Bray–Curtis (abundance) distances (Figure 4). In the Jaccard ordination (Figure 4A), PC1 and PC2 explained 24.9% and 18.9% of total variance, respectively, and sample points were tightly clustered with no host-specific separation. The Bray–Curtis ordination (Figure 4B) yielded greater explanatory power with PC1 at 56.0% and PC2 at 36.5%; still, most samples overlapped substantially, with only minor divergence of Sample 1 and Sample 6.

**Figure 5:**
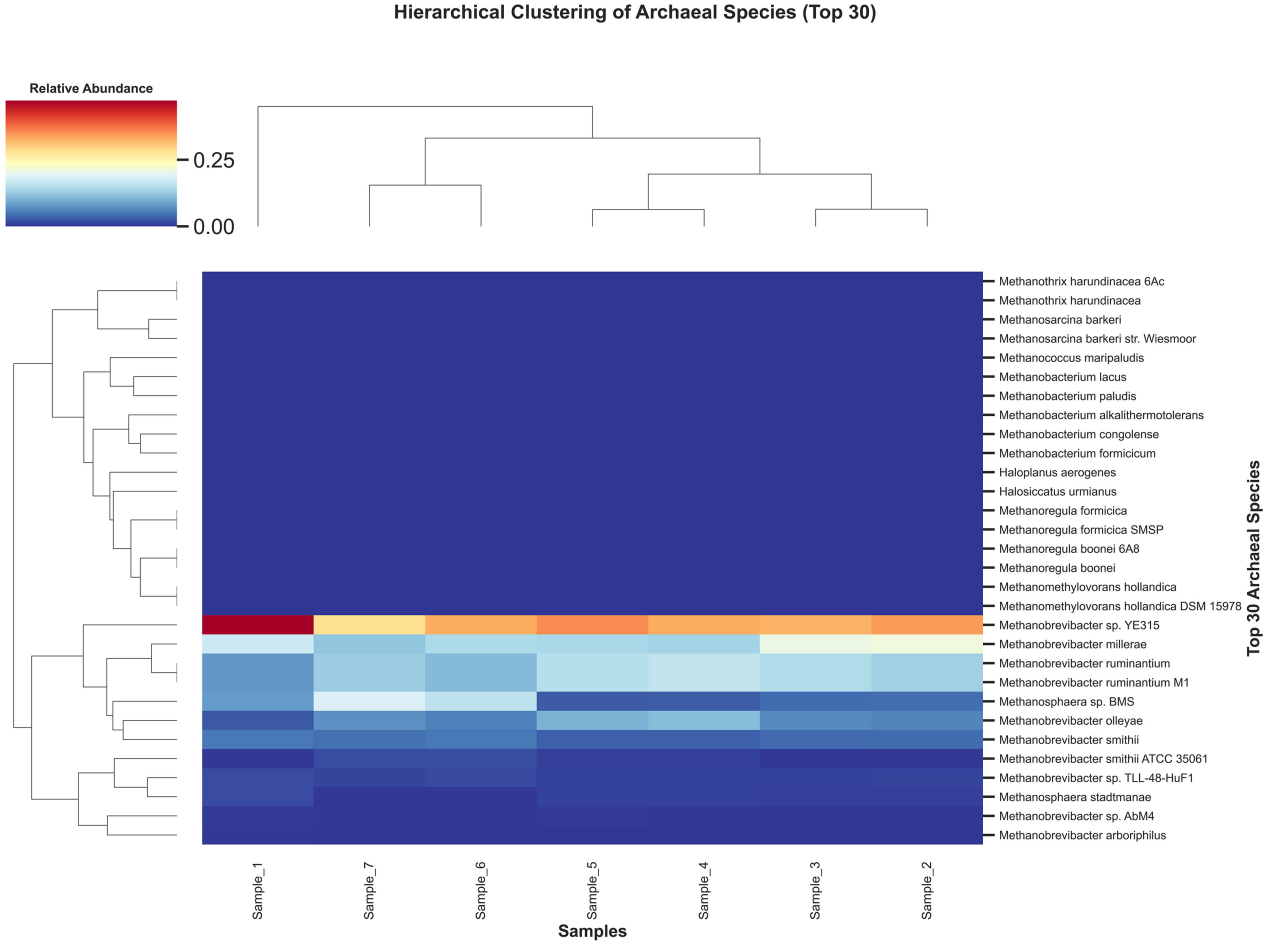
Heatmap and hierarchical clustering of the top 30 archaeal species across camel rumen samples. Species and samples are clustered using Euclidean distance and Ward's linkage; color indicates log10-transformed relative abundance.

Hierarchical clustering of the top 30 archaeal species further illustrated the structural consistency of the archaeal community (Figure 5). The heatmap shows two principal clusters: one dominated by members of the genus *Methanobrevibacter* (e.g., *M. millerae, M. sp. YE315, M. ruminantium*) with high relative abundances across all samples, and a second cluster comprising a suite of low-abundance taxa (e.g., *Methanosphaera* sp., *Methanobacterium* spp.) present at < 1 % relative abundance.

### Core archaeal microbiome

**Figure 6:**
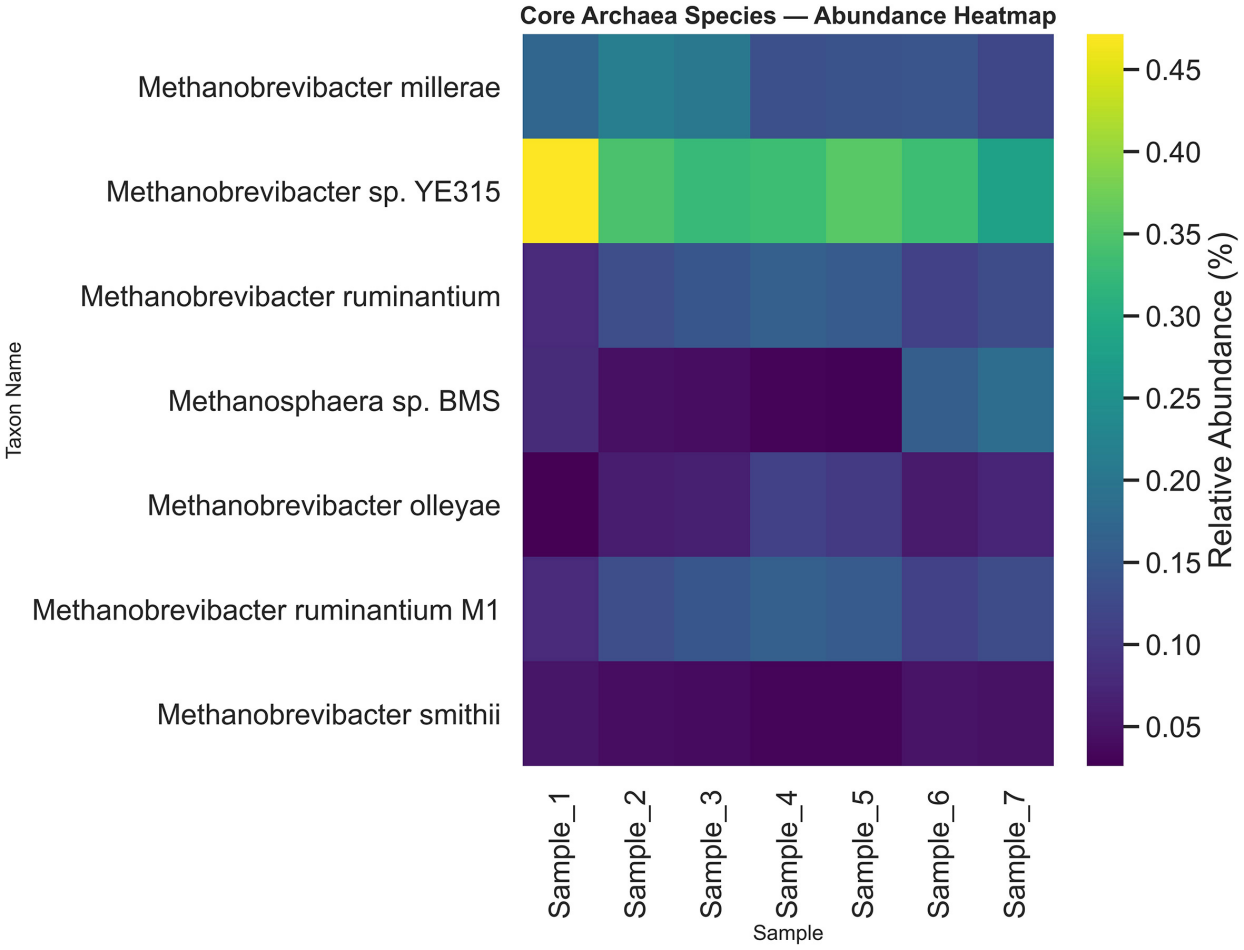
Heatmap and hierarchical clustering of archaeal core species across camel rumen samples. Rows represent individual species, columns correspond to each sample, and cell colors indicate log10-transformed relative abundance values.

**Figure 7:**
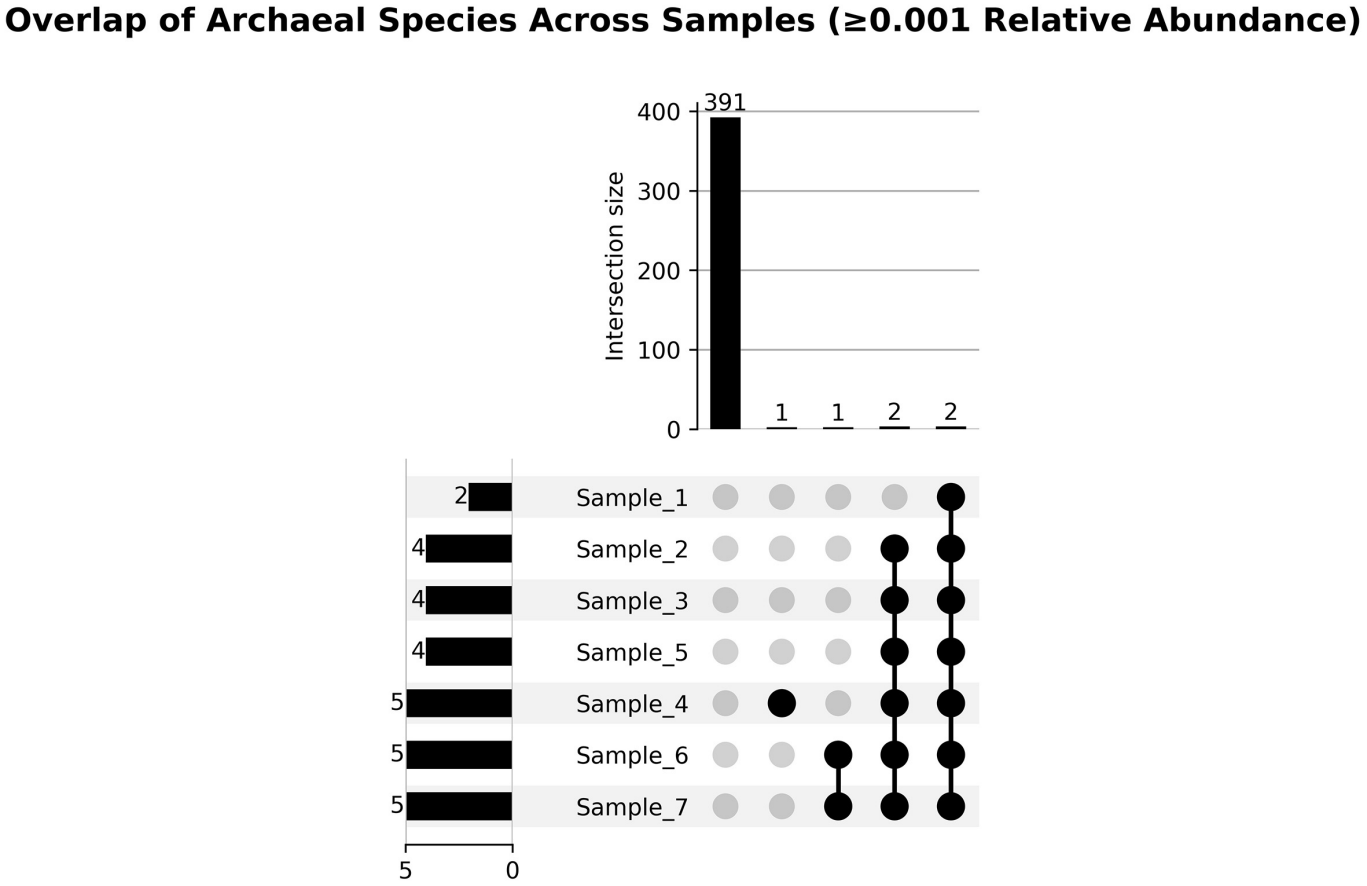
Upset plot showing archaeal species overlap across camel rumen samples. Horizontal bars on the left represent the total number of archaeal species detected in each individual sample. The lower panel displays specific intersections of samples, where connected black dots indicate which samples are included in each intersection. The vertical bars above these intersections represent the number of archaeal species shared by those exact sample combinations. Numbers above the bars correspond to the count of species present in each intersection.

**Figure 8:**
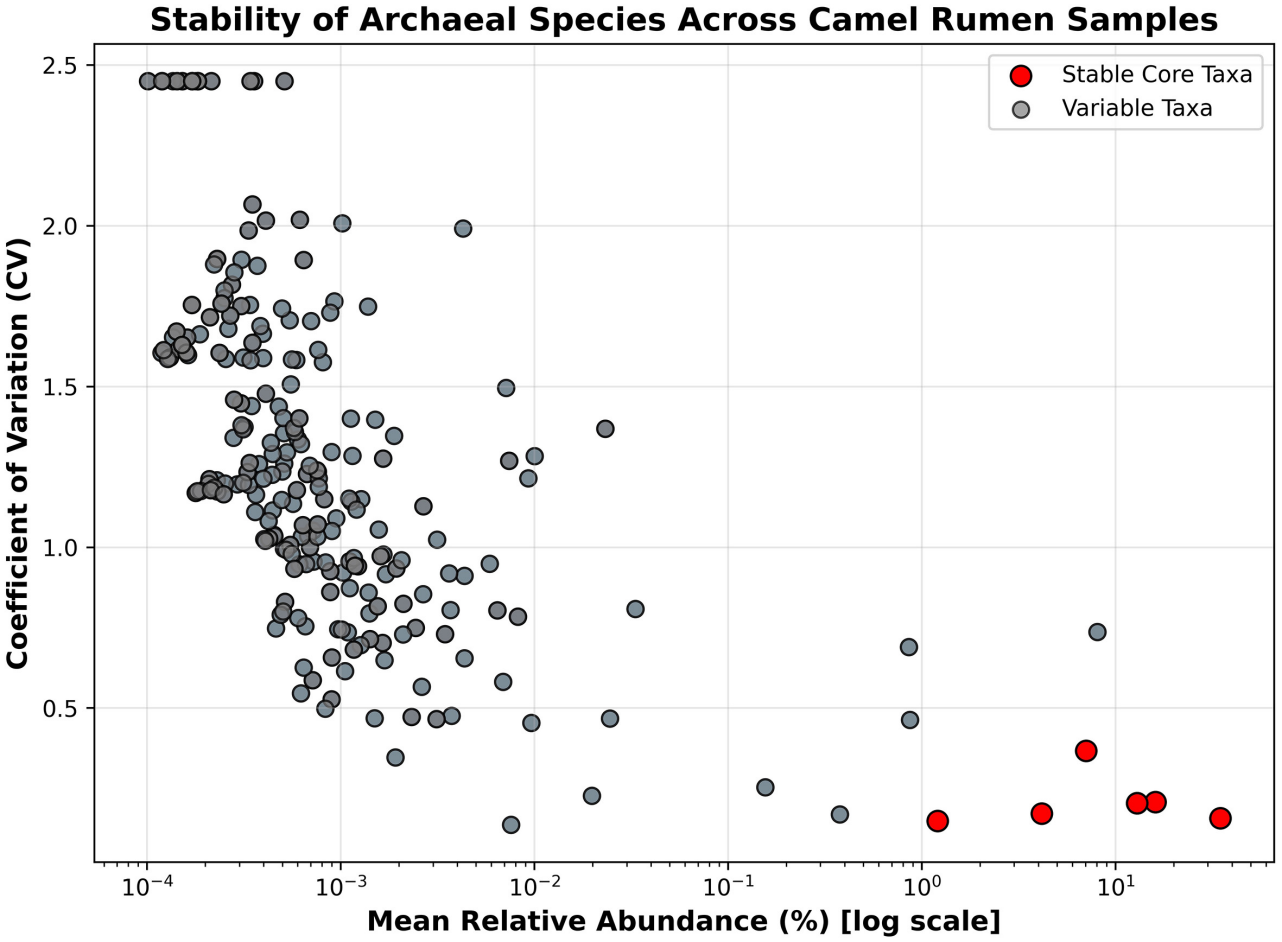
Stability scatterplot of archaeal species mean relative abundance versus coefficient of variation (CV) across camel rumen samples. Each point represents one species; red points represent species present in ≥80 % of samples and are considered core taxa, while gray points represent species with higher variability and/or lower abundance.

Core archaeal taxa were defined as those present in ≥80% of camel rumen samples with a relative abundance ≥0.01%. Based on this criterion, seven dominant archaeal species constituted the core archaeome across all samples (Figure 6, Supplementary Table S2). These taxa included *Methanobrevibacter millerae, Methanobrevibacter* sp. *YE315, Methanobrevibacter ruminantium, Methanosphaera sp. BMS, Methanobrevibacter olleyae, Methanobrevibacter ruminantium M1*, and *Methanobrevibacter smithii*. The Upset-style overlap analysis illustrates that a large pool of archaeal species (391 species-level clades) meets the prevalence threshold, emphasizing a common shared archaeal community across hosts, with very few taxa unique to specific sample subsets (Figure 7). A stability plot further assessed each species' mean relative abundance versus its coefficient of variation (CV) across all samples (Figure 8, Supplementary Table S3). The stability plot shows that dominant taxa such as *Methanobrevibacter sp. YE315* and *Methanobrevibacter millerae* cluster at high mean abundance and low coefficient of variation, thus reinforcing their core classification.

### Functional profile of the archaeal community

**Figure 9:**
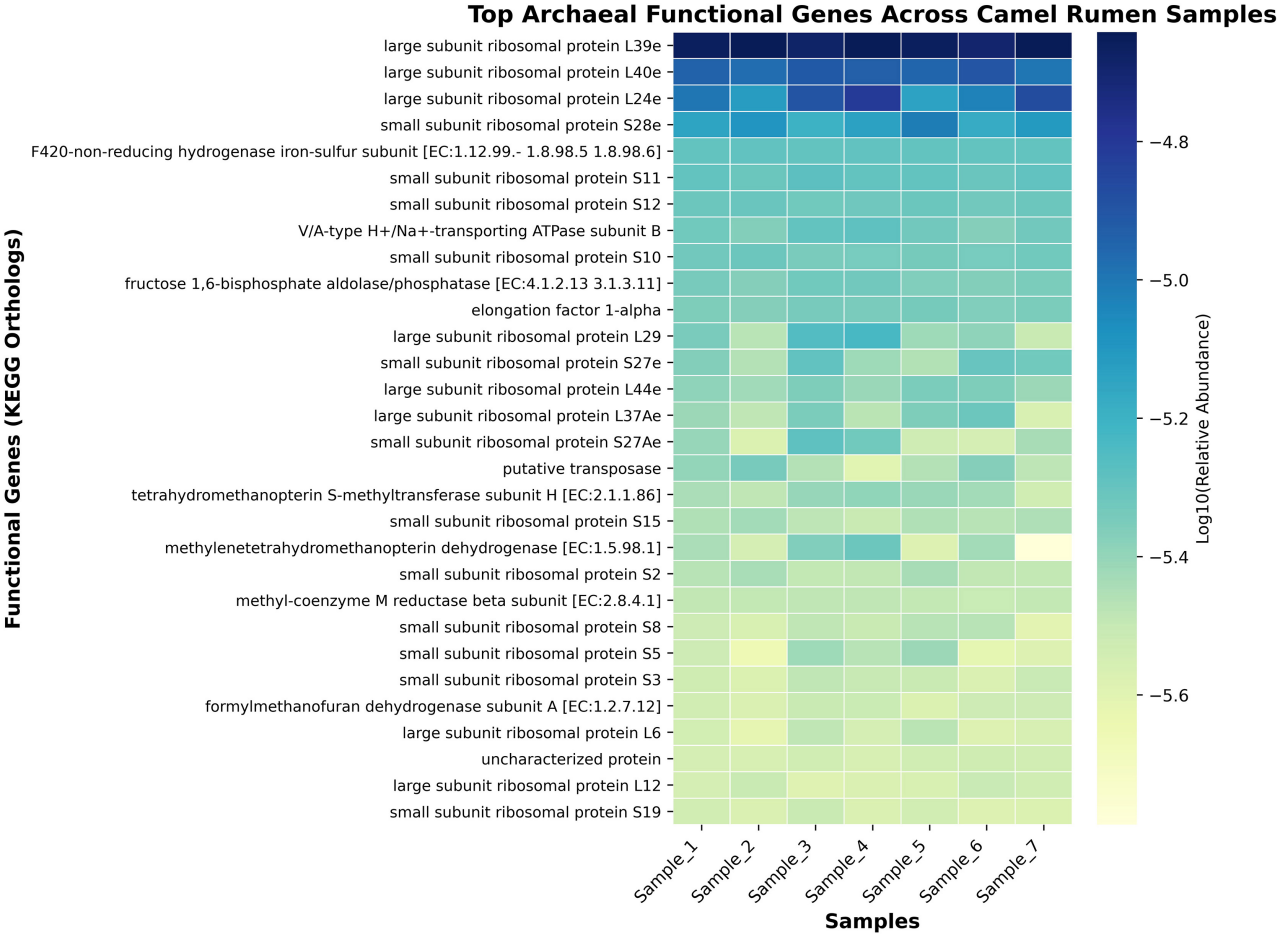
Heatmap of the top archaeal functional genes (KEGG orthologs) identified across camel rumen samples. The color scale represents the log10-transformed relative abundance of each gene.

Shotgun metagenomic functional profiling revealed a diverse repertoire of archaeal functional genes across all camel rumen samples. Functional annotation identified numerous core metabolic and translational processes, predominantly associated with ribosomal protein synthesis, energy metabolism, and methanogenesis (Figure 9, Supplementary Table S4). The most abundant functional profiles across all samples were ribosomal structural proteins, including *large subunit ribosomal proteins* L24e, L39e, L40e, and L29, and *small subunit ribosomal proteins* S10, S11, S12, S27e, and S28e. Genes encoding key enzymes in methanogenesis such as F4_2_0-non-reducing hydrogenase iron-sulfur subunits (EC 1.12.99–1.8.98.6) and methyl-coenzyme M reductase subunits (EC 2.8.4.1) were also detected.

**Figure 10:**
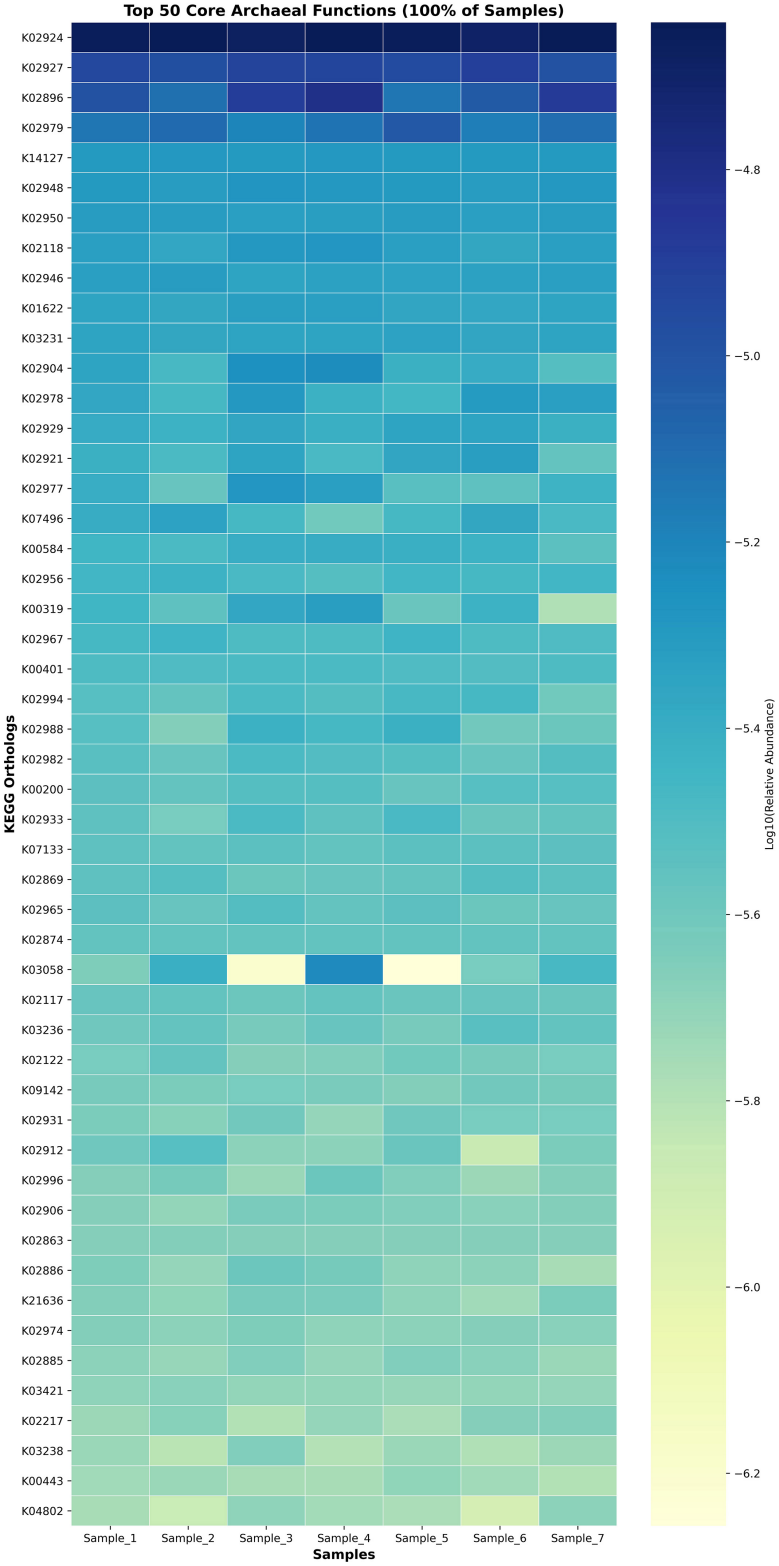
Heatmap of the top 50 core archaeal functions detected across all camel rumen samples. Each cell represents the log10-transformed relative abundance of KEGG orthologs shared by 100% of samples.

To identify the conserved metabolic capacities within the archaeal community of camel rumen samples, we analyzed the core archaeal functions, defined as KEGG Orthologs (KOs) present in 100% of samples. A total of 289 core KOs were detected, indicating a stable set of functional genes maintained across the archaeal population (Figure 10, Supplementary Table S5). The dominant functions were primarily related to translation machinery and energy metabolism, reflecting essential cellular processes necessary for archaeal survival in the rumen environment. Notably, ribosomal protein–encoding genes represented the majority of the core functions, including large subunit ribosomal proteins (K02924, K02927, K02896, K02948, K02950, K02978, and K02979) and small subunit ribosomal proteins (K02977, K02981, K02983, and K02984), suggesting a strong transcriptional and translational stability across individuals. Energy-related enzymes such as F420-non-reducing hydrogenase (K14127), methyl-coenzyme M reductase beta subunit (K00319), and formylmethanofuran dehydrogenase subunit A (K00200) were also present in all samples, indicating the persistence of methanogenesis and hydrogenotrophic energy pathways as core archaeal functions in the camel rumen.

### Functional pathway profiles of the archaeal community

**Figure 11:**
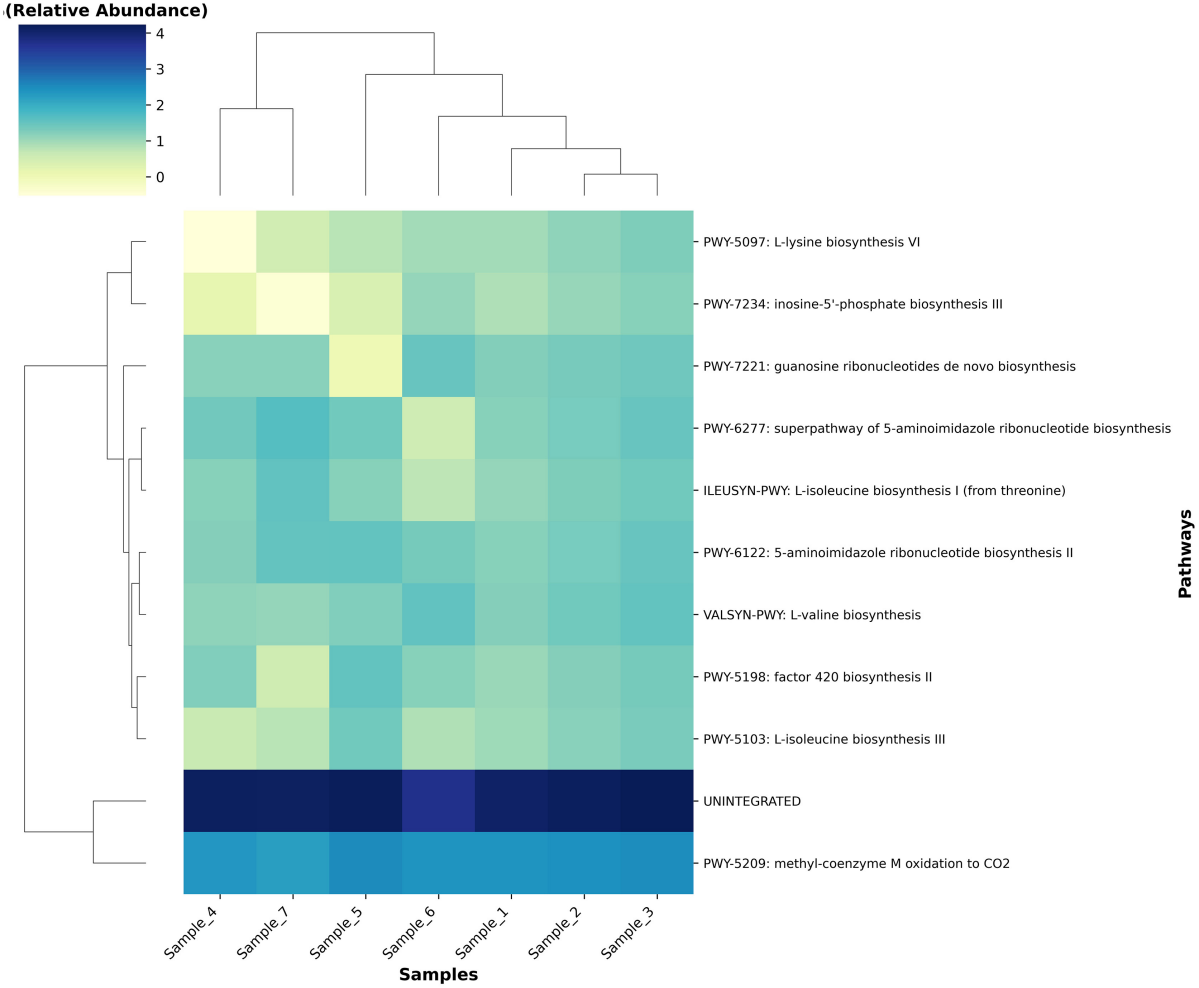
Heatmap and hierarchical clustering of archaeal metabolic pathways across camel rumen samples. Relative abundances (log10-scaled) of annotated pathways (rows) are shown for each sample (columns). Samples are clustered using Euclidean distance and Ward's linkage, and pathways are ordered according to clustering on the left axis. The color bar indicates log10(relative abundance).

Functional pathway reconstruction of the archaeal metagenome revealed key metabolic processes predominantly associated with methanogenic activity and amino acid biosynthesis (Figure 11). The pathway *methyl-coenzyme M oxidation to CO*_2_ *(PWY-5209)* exhibited the highest abundance across all samples (mean = 260.8 ± 59.3). Several amino acid biosynthetic pathways were also highly represented including *L-isoleucine biosynthesis I (ILEUSYN-PWY)* (mean = 18.3 ± 10.5), *L-valine biosynthesis (VALSYN-PWY)* (mean = 22.13 ± 8.8), and *L-lysine biosynthesis VI (PWY-5097)* (mean = 8.7 ± 6.3). Purine nucleotide biosynthetic routes such as *5-aminoimidazole ribonucleotide biosynthesis II (PWY-6122)* (mean = 25.06 ± 10.2), *superpathway of 5-aminoimidazole ribonucleotide biosynthesis (PWY-6277)* (mean = 24.18 ± 10.6), *guanosine ribonucleotides de novo biosynthesis (PWY-7221)* (mean = 18.1 ± 8.4), and *inosine5*′*-phosphate biosynthesis III (PWY-7234)* (mean = 7.4 ± 5.5) were consistently detected, reflecting the active nucleotide metabolism of rumen archaea. Additionally, *factor 420 biosynthesis II (PWY-5198)* (mean = 17.3± 9.9) was identified, a pathway critical for electron transfer in methanogenic archaea.

### Functional diversity of archaeal communities

**Figure 12:**
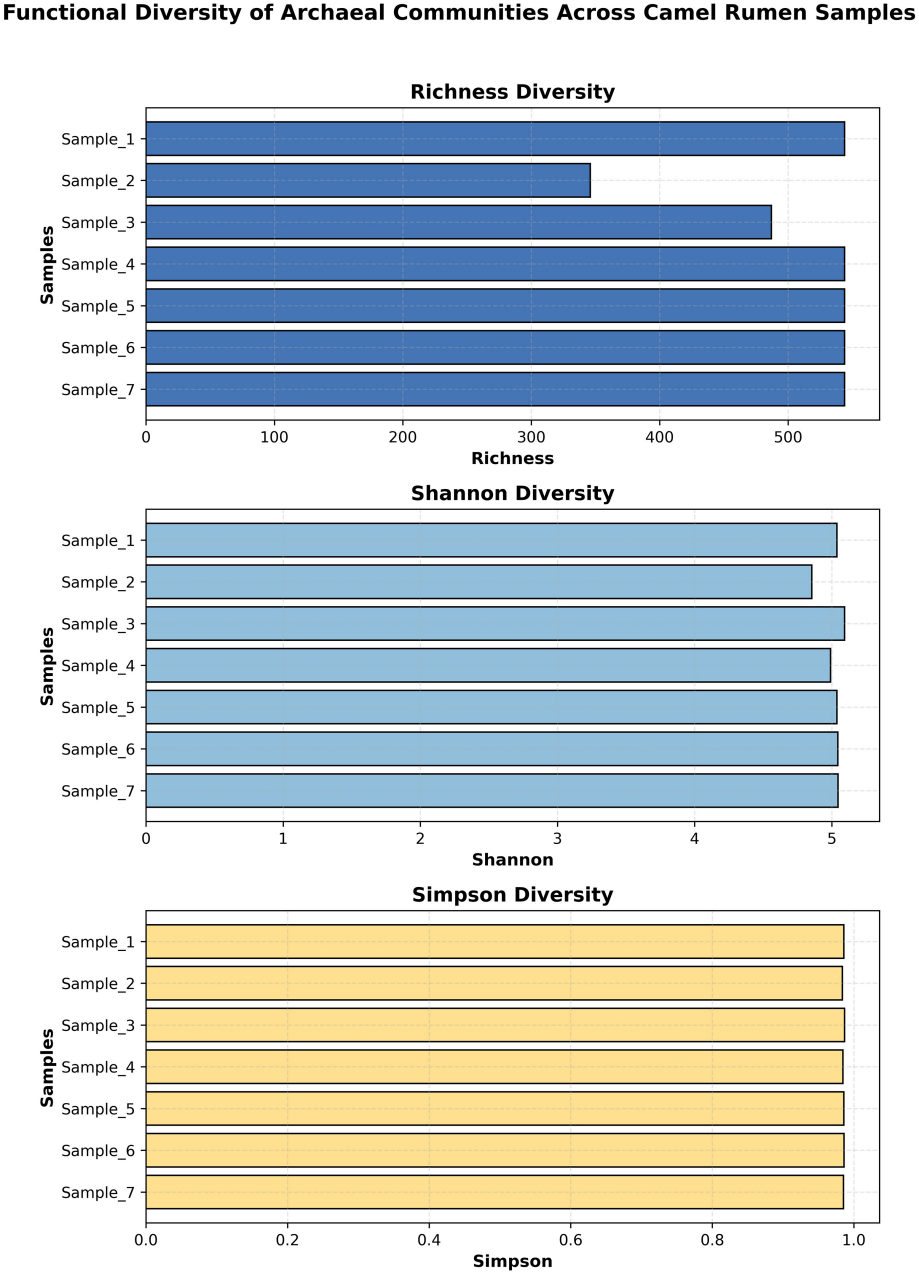
Functional diversity of archaeal communities across camel rumen samples. Horizontal bar-plots show (top panel) richness (number of functional genes), (middle panel) Shannon diversity, and (bottom panel) Simpson diversity indices for each of seven samples. Data are expressed as mean ± standard error (SE).

**Figure 13:**
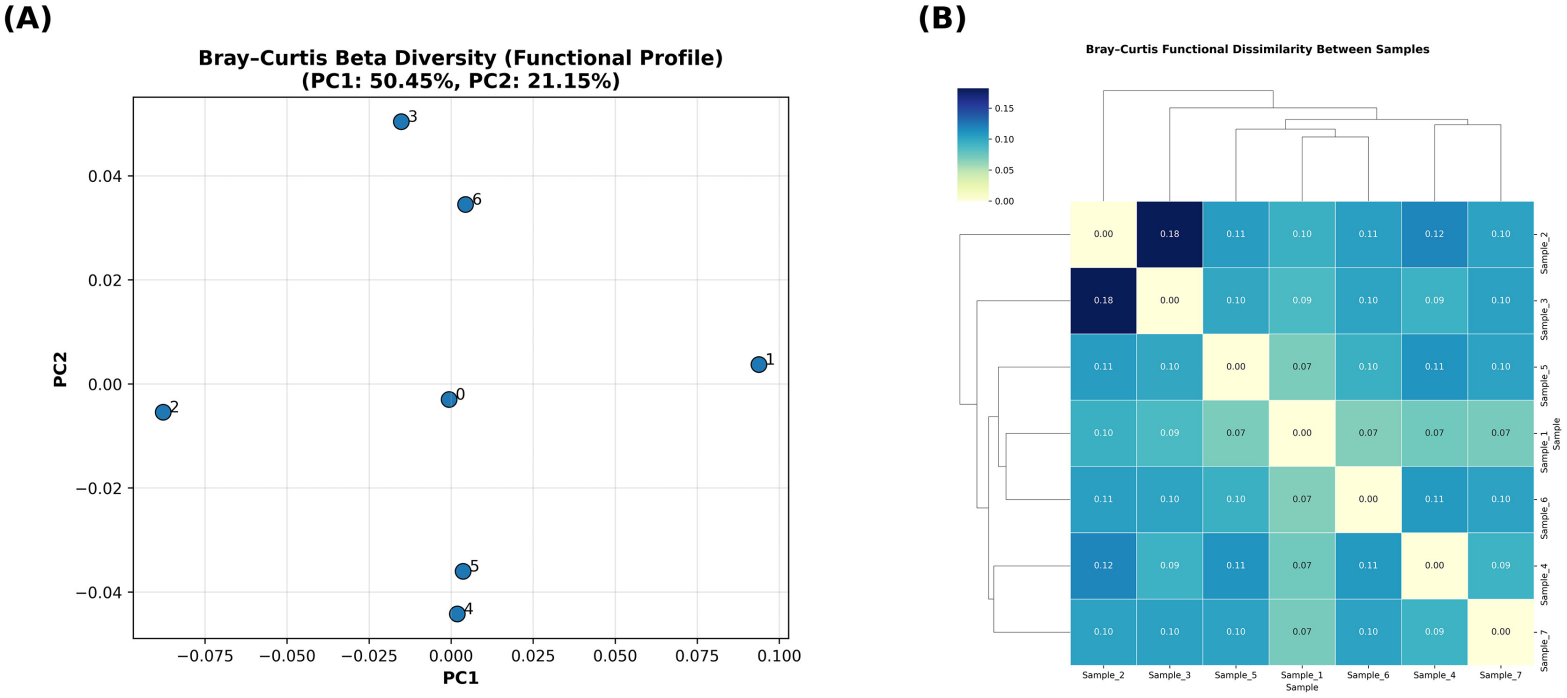
Functional beta diversity of archaeal communities across camel rumen samples. **(A)** Principal coordinate analysis (PCoA) based on Bray–Curtis dissimilarity of archaeal functional gene profiles. **(B)** Hierarchical clustering heatmap representing pairwise Bray–Curtis dissimilarities among samples.

The functional diversity of archaeal communities in the camel rumen, evaluated via richness, Shannon and Simpson indices, revealed a stable gene-repertoire across all samples (mean ± SE: richness = 507 ± 29, Shannon = 5.03 ± 0.05, Simpson = 0.98 ± 0.002) (Figure 12). The functional beta diversity of archaeal communities across the camel rumen samples was examined using Bray–Curtis dissimilarity metrics derived from KEGG ortholog abundance data. Principal coordinate analysis (Figure 13A) revealed a clear clustering pattern among samples, with the first two axes explaining 50.45% and 21.15% of the total variance, respectively. The ordination plot indicated moderate functional differentiation between samples. The hierarchical clustering heatmap of Bray–Curtis dissimilarities (Figure 13B) further supported these findings, illustrating a generally conserved functional landscape among samples, with only subtle variation in pairwise distances (range = 0.07–0.18).

## Discussion

Methanogenic archaea play an important role in the rumen ecosystem by mediating hydrogen turnover through methanogenesis, thereby sustaining the anaerobic conditions necessary for efficient fiber degradation (6). While the rumen archaeome of ruminants has been extensively characterized (28, 29), limited information exists on the archaeal populations inhabiting the camel rumen. Recent advances in WGS metagenomics have enabled simultaneous exploration of both taxonomic and functional features of microbial communities with species-level resolution (11, 28). The current study used metagenomic sequencing to characterize the archaeal composition, diversity, and functional potential of the camel rumen ecosystem. Our findings reveal a conserved and metabolically active archaeome dominated by methanogenic archaea, which plays a central role in hydrogen utilization and methane production within the camel rumen ecosystem. The camel rumen archaeome exhibited a highly conserved taxonomic structure across all seven animals, with Euryarchaeota and Methanomada representing the dominant phyla. This composition aligns with previous studies in ruminants, where Euryarchaeota consistently dominates the archaeal community (6, 28, 29). However, the remarkable stability observed in our study (CV < 5% for dominant taxa) suggests that the camel rumen provides a particularly stable environment for archaeal colonization, potentially due to the unique physiological adaptations of camels to arid environments (30). Within Euryarchaeota, the high abundance of *Methanobacteriaceae* at the family level, particularly *Methanobrevibacter* species, is consistent with findings in other ruminants but shows even greater dominance than typically observed in cattle or sheep (31, 32). This suggests that the camel rumen environment may be particularly favorable for hydrogenotrophic methanogens, which utilize CO_2_ and H_2_ as substrates for methane production (33). The presence of *Methanosphaera* as the second most abundant genus is noteworthy, as this genus is known for its unique methylotrophic metabolism, utilizing methanol and hydrogen for methane production (34). Comparative studies across ruminant species have shown that while core archaeal lineages are shared, host-specific differences exist in abundance patterns (28, 35). Recent study showed that dromedary camels under different feeding systems showed distinct archaeal community compositions, with *Candidatus Methanomethylophilus* and Thermoplasmatales-related archaea among dominant groups (8). Our findings of *Methanobrevibacter* dominance align with the broader ruminant archaeome catalog, which identified *Methanobacteriaceae* as one of the most prevalent families across ten ruminant species (28). *Methanobrevibacter millerae* and *Methanobrevibacter* sp. YE315 were identified as the most abundant archaeal species in the camel rumen, providing insights into the methanogenic populations adapted to this unique environment. *M. millerae* has been reported in various ruminants and is recognized for its efficient hydrogen utilization (36, 37). Its abundance in other species, such as dairy cows, is influenced by diet composition, with non-wheat diets favoring its growth (36). The prominence of *Methanobrevibacter* sp. YE315 indicates that the camel rumen may host archaeal strains uniquely adapted to specific physiological and environmental conditions (38).

The definition of a core archaeome consisting of seven dominant *Methanobrevibacter* species present in ≥80% of samples demonstrate the functional redundancy and stability of the methanogenic community. This core microbiome concept is supported by large-scale comparative studies showing that despite host-specific variations, certain archaeal families remain consistently dominant across ruminant species (28). The low coefficient of variation for these core species indicates remarkable stability, which may be attributed to the consistent diet and management practices of the study animals, as well as the inherent stability of the camel rumen environment (39). Research in other ruminants has shown similar patterns of core archaeal taxa. In sheep, *Methanobrevibacter* was identified as the most dominant genus across different dietary fiber ratios, with *M. ruminantium* showing diet-dependent variation (40). The consistency of *Methanobrevibacter* dominance across ruminant species, including our camel study, reinforces the fundamental role of this genus in ruminant methanogenesis (28).

The functional profiling revealed a metabolic architecture heavily centered on methanogenesis, with the methyl-coenzyme M oxidation pathway (PWY-5209) being the most abundant functional pathway. This finding confirms the primary role of the archaeal community in terminal methane production within the camel rumen (41). The consistent detection of key methanogenic enzymes, including F420-non-reducing hydrogenase and methyl-coenzyme M reductase subunits, across all samples indicates a stable and active methanogenic machinery. Studies linking archaeal gene abundance to methane emissions have demonstrated that methanogenesis genes, particularly mcrA encoding methyl-coenzyme M reductase, correlate significantly with measured methane emissions in cattle (32). The high abundance of these functional genes in our camel samples suggests substantial methanogenic potential, though direct emission measurements would be needed for confirmation. The identification of core KEGG Orthologs (KOs) in 100% of samples, primarily related to translation machinery and energy metabolism, suggests a highly conserved functional core that is essential for archaeal survival and activity in the rumen environment (42). This functional stability, combined with the taxonomic stability, indicates that the camel archaeome represents a well-adapted and specialized microbial community. Comparative metagenomic studies have identified similar functional conservation in hydrogenotrophic methanogens across diverse anaerobic environments (34). The prominence of amino acid biosynthesis pathways (L-isoleucine, L-valine, L-lysine biosynthesis) in the functional profile suggests that archaeal species contribute not only to methane production but also to the overall nitrogen metabolism within the rumen ecosystem (43, 44). Additionally, the detection of Factor 420 biosynthesis (PWY-5198), a cofactor essential for electron transfer in methanogenic archaea, highlights their active role in methanogenesis and energy conservation. Factor 420 mediates electron transfer in key methanogenic enzymes, including F4_2_0-dependent hydrogenases and methyl-coenzyme M reductases, thereby facilitating efficient energy conservation and methane formation (44). This dual functional potential supporting both nitrogen metabolism and efficient methane production may be particularly important in camels, which are adapted to low-quality forage and may rely on microbial protein synthesis to meet their nutritional requirements (45). Although several amino acid biosynthesis pathways were detected in the archaeal metagenome, it is unclear whether rumen archaea actively synthesize these amino acids or if these genes represent remnants of ancestral metabolic capabilities. Therefore, these functional predictions should be interpreted cautiously.

## Study limitations and conclusion

While this study provides a detailed characterization of the camel archaeome and its functional potential, several limitations should be considered when interpreting the findings. All samples were collected from seven clinically healthy racing camels maintained under uniform management and dietary conditions at a single location. Although this controlled setting reduces environmental variability, it also restricts the generalizability of the results, as archaeal communities may differ across regions, diets, production systems, and host genetic backgrounds. Accordingly, our findings should be viewed as descriptive, and future studies involving larger and more diverse camel populations are needed to validate and expand these observations.

Second, the absence of direct methane emission measurements limits our ability to correlate archaeal community composition with actual methane production rates. Therefore, future studies integrating metagenomic data with *in vivo* methane measurements would provide more direct insights into the relationship between archaeal diversity and methane emissions, as demonstrated in other studies (29, 46). Finally, the cross-sectional nature of this study provides a snapshot of the archaeal community at a single time point. Longitudinal studies examining temporal variations in archaeal community structure and function would provide insights into the stability and dynamics of the camel archaeome over time and in response to dietary or environmental changes. In addition, multi-species and broader population studies will be essential to validate and extend these findings.

In conclusion, this study provides a comprehensive metagenomic characterization of the dromedary camel rumen archaeome, revealing a highly stable and conserved community dominated by methanogenic archaea. The identification of a core archaeome, along with their associated functional repertoire, provides a foundation for understanding methane production in camels and developing targeted mitigation strategies.

## Data Availability

The datasets presented in this study can be found in online repositories. The names of the repository/repositories and accession number(s) can be found at: https://www.ncbi.nlm.nih.gov/, PRJNA1377671.

## Supplementary material

Supplementary Table S1 Relative abundance of the archaeal community in camel rumen samples at phylum, family, genus and species level.

Supplementary Table S2 Core archaeal taxa identified in camel rumen samples. Core taxa were defined as those present in ≥80% of samples with a relative abundance ≥0.01%.

Supplementary Table S3 Stability analysis of archaeal species in camel rumen samples. Each species is characterized by its mean relative abundance and coefficient of variation (CV) across all samples.

Supplementary Table S4 Functional annotation of core archaeal genes identified in camel rumen samples.

Supplementary Table S5 Core archaeal functions identified in camel rumen samples. Core functions were defined as KEGG Orthologs (KOs) detected in 100% of samples.
